# Dinitro­sylbis[tris­(4-fluoro­phen­yl)phosphane]iron chloro­form monosolvate

**DOI:** 10.1107/S1600536811004673

**Published:** 2011-02-12

**Authors:** Myron W. Jones, Douglas R. Powell, George B. Richter-Addo

**Affiliations:** a Department of Chemistry and Biochemistry, University of Oklahoma, 101 Stephenson Parkway, Norman, OK 73019-5251, USA

## Abstract

The title compound, [Fe(NO)_2_(C_18_H_12_F_3_P)_2_]·CHCl_3_, belongs to the family of metal dinitrosyl compounds with the general formula Fe(NO)_2_(*L*)_*x*_, referred to collectively as ‘dinitrosyl iron compounds’ (DNICs). Herein we report the structure of a dinitrosyl iron diphosphane complex, (Fe(NO)_2_ *L*_2_, with *L* = P(C_6_H_4_-*p*-F)_3_. The structure includes one metal complex mol­ecule and one chloro­form solvent mol­ecule. The iron atom is tetra­hedrally coordinated with two phosphane ligands and with two NO groups with Fe—N—O angles of 178.1 (2) and 177.0 (2)°.

## Related literature

The starting compound, Fe(NO)_2_(CO)_2_, was prepared using a published method described by Eisch & King (1965 ▶). For the structures of some related dinitrosyl complexes, see: Li *et al.* (2003 ▶); Atkinson *et al.* (1996 ▶); Li Kam Wah *et al.* (1989 ▶); Albano *et al.* (1974 ▶). For general information on metal nitrosyl chemistry, see: Richter-Addo & Legzdins (1992 ▶).
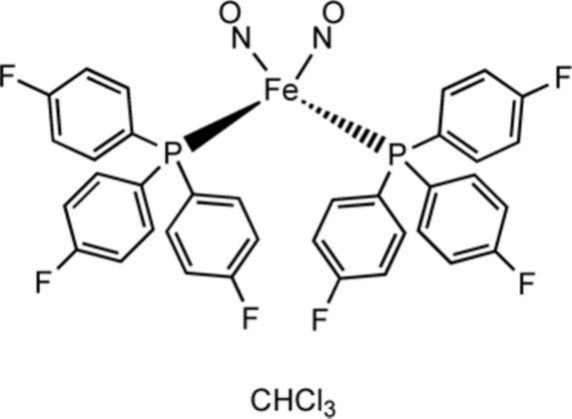


## Experimental

### 

#### Crystal data

[Fe(NO)_2_(C_18_H_12_F_3_P)_2_]·CHCl_3_
*M* *_r_* = 867.73
Monoclinic, 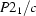

*a* = 13.994 (5) Å
*b* = 15.746 (6) Å
*c* = 16.716 (6) Å
β = 97.651 (8)°
*V* = 3651 (2) Å^3^
*Z* = 4
Mo *K*α radiation
μ = 0.79 mm^−1^
*T* = 100 K
0.44 × 0.22 × 0.04 mm

#### Data collection

Bruker APEX CCD diffractometer
Absorption correction: multi-scan (*SADABS*; Sheldrick, 2001 ▶) *T*_min_ = 0.721, *T*_max_ = 0.972
22634 measured reflections
6325 independent reflections
5006 reflections with *I* > 2σ(*I*)
*R*_int_ = 0.056

#### Refinement

*R*[*F* ^2^ > 2σ(*F* ^2^)] = 0.042
*wR*(*F* ^2^) = 0.115
*S* = 1.02
6325 reflections
478 parameters
H-atom parameters constrained
Δρ_max_ = 1.24 e Å^−3^
Δρ_min_ = −0.46 e Å^−3^

### 

Data collection: *SMART* (Bruker, 2007 ▶); cell refinement: *SAINT* (Bruker, 2007 ▶); data reduction: *SAINT*; program(s) used to solve structure: *SHELXTL* (Sheldrick, 2008 ▶); program(s) used to refine structure: *SHELXTL*; molecular graphics: *SHELXTL*; software used to prepare material for publication: *SHELXTL*.

## Supplementary Material

Crystal structure: contains datablocks I, global. DOI: 10.1107/S1600536811004673/fk2035sup1.cif

Structure factors: contains datablocks I. DOI: 10.1107/S1600536811004673/fk2035Isup2.hkl

Additional supplementary materials:  crystallographic information; 3D view; checkCIF report

## Figures and Tables

**Fig. 1.**
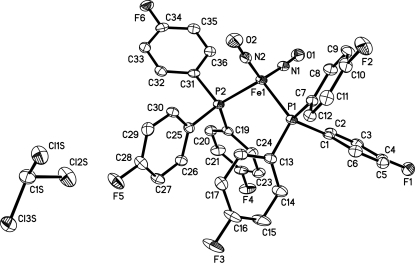
The molecular structure of the title compound. Hydrogen atoms were omitted for clarity. The displacement ellipsoids were drawn at the 50% probability level.

## supplementary crystallographic information

### Comment

The molecular structure of the title compound is shown in Fig. 1. The structure includes one metal complex molecule and one chloroform solvent molecule. The metal complex molecule possesses a distorted tetrahedral geometry around the iron center. The iron is bound to two nitrosyl groups *via* the nitrogen atoms and to two phosphine ligands *via* the phorphorus atoms. The Fe(NO)_2_ moiety exhibits an *attracto* conformation where the bond angle O···Fe···O < N—Fe—N (Richter-Addo & Legzdins, 1992). The N—Fe—N bond angle is 127.02 (11)° and the interphosphine bond angle, P—Fe—P, is 108.27 (4)°. The Fe—N—O bond angles are 178.1 (2)° and 177.0 (2)°.

### Experimental

A light yellow toluene solution (5 ml) of P(C_6_H_4_-*p*-F)_3_ (127 mg, 0.40 mmol) was charged with Fe(NO)_2_(CO)_2_ (21 µ*L*, 0.19 mmol) (Eisch & King, 1965). The light red/orange solution was heated and stirred under nitrogen for 3.25 h after which time the infrared spectrum was consistent with the presence of the product and no trace of Fe(NO)_2_(CO)_2_ (ν_CO_ = 2090 cm^-1^ and 2040 cm^-1^) was observed. The reaction mixture was filtered through celite under N_2_ and the solvent was subsequently removed under vacuum. Isolated yield of the Fe(NO)_2_*L*_2_ compound: 23%. IR (toluene, cm^-1^): ν_NO_ = 1720 s and 1682 s; ^31^P{^1^H} NMR (CDCl_3_): δ 59.3 (*s*) referenced to 85% H_3_PO_4_. Suitable crystals for X-ray diffraction studies were grown by slow evaporation of a chloroform solution of the complex under nitrogen at ambient temperature.

### Refinement

H atoms were placed using known geometry with C—H (phenyl = 0.95 Å, methylene = 1.00 Å). Displacement parameters of phenyl H atoms were set to 1.2 times the isotropic equivalent for the bonded C.

### Crystal data

**Table:**

[Fe(NO)_2_(C_18_H_12_F_3_P)_2_]·CHCl_3_	*F*(000) = 1752
*M**_r_* = 867.73	*D*_x_ = 1.579 Mg m^−^^3^
Monoclinic, *P*2_1_/*c*	Mo *K*α radiation, λ = 0.71073 Å
Hall symbol: -P 2ybc	Cell parameters from 5630 reflections
*a* = 13.994 (5) Å	θ = 2.4–25.7°
*b* = 15.746 (6) Å	µ = 0.79 mm^−^^1^
*c* = 16.716 (6) Å	*T* = 100 K
β = 97.651 (8)°	Prism, red
*V* = 3651 (2) Å^3^	0.44 × 0.22 × 0.04 mm
*Z* = 4	

### Data collection

**Table:**

Bruker APEX CCD diffractometer	6325 independent reflections
Radiation source: fine-focus sealed tube	5006 reflections with *I* > 2σ(*I*)
graphite	*R*_int_ = 0.056
ω scans	θ_max_ = 25.0°, θ_min_ = 2.0°
Absorption correction: multi-scan (*SADABS*; Sheldrick, 2001)	*h* = −16→16
*T*_min_ = 0.721, *T*_max_ = 0.972	*k* = −18→18
22634 measured reflections	*l* = −19→19

### Refinement

**Table:**

Refinement on *F*^2^	Primary atom site location: structure-invariant direct methods
Least-squares matrix: full	Secondary atom site location: difference Fourier map
*R*[*F*^2^ > 2σ(*F*^2^)] = 0.042	Hydrogen site location: inferred from neighbouring sites
*wR*(*F*^2^) = 0.115	H-atom parameters constrained
*S* = 1.02	*w* = 1/[σ^2^(*F*_o_^2^) + (0.074*P*)^2^] where *P* = (*F*_o_^2^ + 2*F*_c_^2^)/3
6325 reflections	(Δ/σ)_max_ = 0.001
478 parameters	Δρ_max_ = 1.24 e Å^−^^3^
0 restraints	Δρ_min_ = −0.46 e Å^−^^3^

### Fractional atomic coordinates and isotropic or equivalent isotropic displacement parameters (Å^2^)

**Table:**

	*x*	*y*	*z*	*U*_iso_*/*U*_eq_	
Fe1	0.37827 (3)	0.78849 (2)	0.33064 (2)	0.01773 (13)	
P1	0.47836 (5)	0.73871 (4)	0.24750 (4)	0.01786 (18)	
P2	0.22602 (5)	0.76296 (4)	0.27435 (4)	0.01742 (18)	
F1	0.64936 (13)	0.98173 (11)	0.03475 (10)	0.0316 (4)	
F2	0.83075 (13)	0.62487 (11)	0.45867 (11)	0.0377 (5)	
F3	0.34188 (16)	0.44281 (11)	0.04389 (11)	0.0474 (6)	
F4	0.08751 (14)	0.94290 (11)	−0.03130 (9)	0.0358 (5)	
F5	0.11645 (14)	0.40322 (10)	0.20664 (11)	0.0380 (5)	
F6	−0.03371 (12)	0.88266 (11)	0.50522 (10)	0.0325 (4)	
O1	0.39333 (16)	0.96864 (13)	0.34007 (12)	0.0307 (5)	
O2	0.41657 (17)	0.67401 (13)	0.46305 (13)	0.0366 (6)	
N1	0.38753 (17)	0.89340 (15)	0.33402 (13)	0.0213 (5)	
N2	0.39889 (17)	0.72178 (14)	0.40736 (14)	0.0228 (5)	
C1	0.5291 (2)	0.81458 (17)	0.18170 (16)	0.0185 (6)	
C2	0.4853 (2)	0.89178 (16)	0.16116 (16)	0.0194 (6)	
H2	0.4273	0.9063	0.1818	0.023*	
C3	0.5248 (2)	0.94872 (17)	0.11063 (17)	0.0223 (6)	
H3	0.4943	1.0014	0.0961	0.027*	
C4	0.6085 (2)	0.92645 (18)	0.08276 (16)	0.0227 (6)	
C5	0.6549 (2)	0.85066 (18)	0.10161 (17)	0.0246 (7)	
H5	0.7132	0.8373	0.0811	0.030*	
C6	0.6146 (2)	0.79450 (18)	0.15110 (17)	0.0236 (7)	
H6	0.6453	0.7416	0.1645	0.028*	
C7	0.5880 (2)	0.69932 (17)	0.30903 (16)	0.0195 (6)	
C8	0.6333 (2)	0.75458 (18)	0.36730 (17)	0.0244 (7)	
H8	0.6068	0.8094	0.3733	0.029*	
C9	0.7164 (2)	0.73055 (19)	0.41664 (18)	0.0273 (7)	
H9	0.7489	0.7689	0.4549	0.033*	
C10	0.7504 (2)	0.64994 (19)	0.40848 (17)	0.0258 (7)	
C11	0.7081 (2)	0.59301 (18)	0.35257 (18)	0.0250 (7)	
H11	0.7341	0.5377	0.3486	0.030*	
C12	0.6262 (2)	0.61843 (18)	0.30184 (17)	0.0227 (6)	
H12	0.5962	0.5804	0.2621	0.027*	
C13	0.4398 (2)	0.64918 (17)	0.18126 (16)	0.0200 (6)	
C14	0.4392 (2)	0.65064 (19)	0.09849 (17)	0.0295 (7)	
H14	0.4615	0.6996	0.0735	0.035*	
C15	0.4061 (3)	0.5809 (2)	0.05134 (19)	0.0380 (8)	
H15	0.4056	0.5816	−0.0055	0.046*	
C16	0.3742 (2)	0.51132 (18)	0.08944 (19)	0.0317 (8)	
C17	0.3733 (2)	0.50733 (17)	0.17158 (17)	0.0233 (6)	
H17	0.3514	0.4579	0.1962	0.028*	
C18	0.4053 (2)	0.57763 (17)	0.21709 (17)	0.0218 (6)	
H18	0.4037	0.5771	0.2737	0.026*	
C19	0.1808 (2)	0.82030 (16)	0.18227 (16)	0.0197 (6)	
C20	0.0885 (2)	0.85538 (18)	0.16838 (17)	0.0243 (7)	
H20	0.0467	0.8507	0.2085	0.029*	
C21	0.0571 (2)	0.89696 (19)	0.09674 (18)	0.0291 (7)	
H21	−0.0056	0.9212	0.0874	0.035*	
C22	0.1186 (2)	0.90232 (18)	0.03967 (16)	0.0251 (7)	
C23	0.2093 (2)	0.86878 (18)	0.04979 (17)	0.0267 (7)	
H23	0.2501	0.8738	0.0089	0.032*	
C24	0.2401 (2)	0.82711 (18)	0.12187 (17)	0.0233 (6)	
H24	0.3028	0.8028	0.1302	0.028*	
C25	0.19084 (19)	0.65336 (17)	0.25032 (16)	0.0191 (6)	
C26	0.1510 (2)	0.62711 (17)	0.17335 (16)	0.0208 (6)	
H26	0.1403	0.6675	0.1309	0.025*	
C27	0.1267 (2)	0.54248 (18)	0.15809 (17)	0.0253 (7)	
H27	0.1007	0.5243	0.1055	0.030*	
C28	0.1411 (2)	0.48607 (18)	0.22068 (18)	0.0265 (7)	
C29	0.1803 (2)	0.50879 (18)	0.29794 (18)	0.0249 (7)	
H29	0.1890	0.4680	0.3402	0.030*	
C30	0.2062 (2)	0.59248 (17)	0.31186 (17)	0.0206 (6)	
H30	0.2350	0.6092	0.3642	0.025*	
C31	0.1431 (2)	0.79517 (17)	0.34546 (16)	0.0188 (6)	
C32	0.0698 (2)	0.74415 (18)	0.36738 (17)	0.0226 (6)	
H32	0.0600	0.6890	0.3447	0.027*	
C33	0.0109 (2)	0.77292 (18)	0.42185 (17)	0.0234 (6)	
H33	−0.0388	0.7380	0.4374	0.028*	
C34	0.0260 (2)	0.85280 (19)	0.45277 (17)	0.0240 (7)	
C35	0.0971 (2)	0.90590 (18)	0.43289 (17)	0.0253 (7)	
H35	0.1051	0.9613	0.4553	0.030*	
C36	0.1564 (2)	0.87624 (18)	0.37938 (17)	0.0237 (6)	
H36	0.2069	0.9113	0.3654	0.028*	
Cl1S	0.22226 (6)	0.10408 (5)	0.38685 (5)	0.0335 (2)	
Cl2S	0.13359 (6)	0.23683 (5)	0.28321 (6)	0.0417 (2)	
Cl3S	0.08805 (6)	0.06170 (5)	0.24465 (5)	0.0344 (2)	
C1S	0.1178 (2)	0.13555 (19)	0.32295 (19)	0.0297 (7)	
H1S	0.0631	0.1383	0.3557	0.036*	

### Atomic displacement parameters (Å^2^)

**Table:**

	*U*^11^	*U*^22^	*U*^33^	*U*^12^	*U*^13^	*U*^23^
Fe1	0.0201 (2)	0.0199 (2)	0.0119 (2)	0.00032 (16)	−0.00246 (16)	−0.00012 (15)
P1	0.0197 (4)	0.0194 (4)	0.0134 (4)	0.0006 (3)	−0.0022 (3)	−0.0002 (3)
P2	0.0198 (4)	0.0196 (4)	0.0118 (4)	0.0009 (3)	−0.0019 (3)	0.0005 (3)
F1	0.0371 (11)	0.0325 (9)	0.0249 (10)	−0.0067 (8)	0.0033 (8)	0.0082 (8)
F2	0.0271 (10)	0.0443 (11)	0.0363 (11)	0.0028 (8)	−0.0156 (8)	0.0083 (9)
F3	0.0810 (16)	0.0270 (10)	0.0271 (10)	−0.0122 (9)	−0.0192 (10)	−0.0062 (8)
F4	0.0521 (12)	0.0392 (10)	0.0137 (9)	0.0141 (9)	−0.0048 (8)	0.0082 (7)
F5	0.0498 (12)	0.0205 (9)	0.0384 (11)	−0.0079 (8)	−0.0137 (9)	0.0008 (8)
F6	0.0336 (11)	0.0411 (11)	0.0245 (10)	0.0029 (8)	0.0098 (8)	−0.0061 (8)
O1	0.0412 (14)	0.0221 (11)	0.0293 (12)	−0.0027 (10)	0.0064 (10)	−0.0002 (9)
O2	0.0474 (15)	0.0364 (12)	0.0228 (12)	−0.0005 (11)	−0.0065 (10)	0.0143 (10)
N1	0.0237 (14)	0.0232 (14)	0.0162 (13)	0.0005 (10)	−0.0005 (10)	−0.0008 (10)
N2	0.0244 (14)	0.0237 (12)	0.0196 (14)	−0.0033 (10)	0.0006 (11)	−0.0007 (10)
C1	0.0223 (16)	0.0218 (14)	0.0102 (14)	−0.0025 (12)	−0.0025 (11)	−0.0029 (11)
C2	0.0203 (15)	0.0229 (14)	0.0136 (14)	0.0015 (12)	−0.0024 (11)	−0.0035 (11)
C3	0.0267 (17)	0.0217 (14)	0.0161 (15)	0.0004 (12)	−0.0054 (12)	0.0010 (12)
C4	0.0286 (17)	0.0266 (15)	0.0115 (14)	−0.0069 (13)	−0.0021 (12)	−0.0006 (12)
C5	0.0241 (17)	0.0294 (16)	0.0201 (16)	−0.0001 (13)	0.0020 (13)	−0.0010 (13)
C6	0.0272 (17)	0.0216 (15)	0.0205 (16)	0.0044 (12)	−0.0013 (13)	0.0030 (12)
C7	0.0187 (15)	0.0233 (14)	0.0153 (15)	0.0000 (12)	−0.0018 (11)	0.0023 (11)
C8	0.0259 (17)	0.0263 (15)	0.0195 (16)	0.0019 (13)	−0.0022 (13)	−0.0006 (12)
C9	0.0263 (17)	0.0336 (17)	0.0194 (16)	−0.0043 (13)	−0.0070 (13)	−0.0016 (13)
C10	0.0181 (16)	0.0358 (17)	0.0211 (16)	0.0007 (13)	−0.0054 (12)	0.0097 (13)
C11	0.0237 (17)	0.0229 (15)	0.0281 (17)	0.0049 (13)	0.0020 (13)	0.0051 (13)
C12	0.0217 (16)	0.0264 (15)	0.0193 (16)	−0.0016 (12)	0.0000 (12)	−0.0011 (12)
C13	0.0199 (15)	0.0212 (14)	0.0174 (15)	0.0032 (12)	−0.0026 (12)	−0.0019 (11)
C14	0.043 (2)	0.0256 (16)	0.0171 (16)	−0.0029 (14)	−0.0052 (14)	0.0025 (12)
C15	0.064 (2)	0.0313 (17)	0.0140 (16)	−0.0036 (16)	−0.0114 (15)	−0.0002 (13)
C16	0.045 (2)	0.0210 (15)	0.0239 (17)	−0.0020 (14)	−0.0135 (14)	−0.0052 (13)
C17	0.0229 (16)	0.0211 (14)	0.0239 (17)	0.0002 (12)	−0.0042 (12)	0.0035 (12)
C18	0.0208 (16)	0.0256 (15)	0.0175 (15)	0.0041 (12)	−0.0026 (12)	−0.0013 (12)
C19	0.0263 (16)	0.0170 (13)	0.0137 (14)	−0.0022 (12)	−0.0048 (12)	0.0010 (11)
C20	0.0246 (17)	0.0269 (15)	0.0210 (16)	0.0032 (13)	0.0009 (12)	−0.0006 (12)
C21	0.0310 (18)	0.0339 (17)	0.0205 (16)	0.0123 (14)	−0.0042 (13)	0.0015 (13)
C22	0.0393 (19)	0.0224 (15)	0.0114 (15)	0.0043 (13)	−0.0043 (13)	0.0016 (11)
C23	0.0354 (19)	0.0306 (16)	0.0135 (15)	−0.0016 (14)	0.0007 (13)	0.0020 (12)
C24	0.0221 (16)	0.0278 (15)	0.0185 (15)	0.0018 (12)	−0.0023 (12)	0.0016 (12)
C25	0.0153 (15)	0.0231 (14)	0.0183 (15)	0.0001 (11)	−0.0004 (11)	−0.0007 (12)
C26	0.0215 (16)	0.0230 (14)	0.0165 (15)	0.0020 (12)	−0.0023 (12)	0.0019 (11)
C27	0.0274 (17)	0.0265 (16)	0.0193 (16)	0.0000 (13)	−0.0067 (13)	−0.0031 (12)
C28	0.0259 (17)	0.0232 (15)	0.0285 (17)	−0.0020 (13)	−0.0032 (13)	−0.0017 (13)
C29	0.0239 (17)	0.0271 (15)	0.0227 (16)	0.0003 (13)	−0.0003 (13)	0.0076 (12)
C30	0.0205 (16)	0.0244 (14)	0.0153 (15)	0.0004 (12)	−0.0036 (12)	−0.0004 (11)
C31	0.0207 (16)	0.0233 (14)	0.0111 (14)	0.0035 (12)	−0.0032 (11)	0.0008 (11)
C32	0.0224 (16)	0.0245 (15)	0.0189 (16)	0.0007 (12)	−0.0047 (12)	0.0003 (12)
C33	0.0210 (16)	0.0282 (16)	0.0199 (16)	−0.0009 (12)	−0.0010 (12)	0.0015 (12)
C34	0.0246 (16)	0.0336 (17)	0.0136 (15)	0.0066 (13)	0.0016 (12)	−0.0002 (12)
C35	0.0294 (17)	0.0239 (15)	0.0222 (16)	0.0014 (13)	0.0017 (13)	−0.0039 (12)
C36	0.0263 (17)	0.0253 (15)	0.0193 (15)	−0.0022 (13)	0.0019 (13)	−0.0016 (12)
Cl1S	0.0312 (5)	0.0322 (4)	0.0338 (5)	−0.0014 (3)	−0.0086 (3)	0.0084 (3)
Cl2S	0.0412 (5)	0.0307 (4)	0.0479 (6)	−0.0011 (4)	−0.0140 (4)	0.0073 (4)
Cl3S	0.0350 (5)	0.0372 (4)	0.0297 (4)	−0.0069 (3)	−0.0005 (3)	−0.0039 (3)
C1S	0.0296 (18)	0.0324 (17)	0.0253 (17)	0.0019 (14)	−0.0027 (13)	0.0001 (14)

### Geometric parameters (Å, °)

**Table:**

Fe1—N2	1.653 (2)	C14—H14	0.9500
Fe1—N1	1.657 (2)	C15—C16	1.371 (4)
Fe1—P1	2.2420 (10)	C15—H15	0.9500
Fe1—P2	2.2478 (11)	C16—C17	1.376 (4)
P1—C13	1.829 (3)	C17—C18	1.383 (4)
P1—C1	1.830 (3)	C17—H17	0.9500
P1—C7	1.837 (3)	C18—H18	0.9500
P2—C19	1.824 (3)	C19—C24	1.394 (4)
P2—C25	1.825 (3)	C19—C20	1.396 (4)
P2—C31	1.840 (3)	C20—C21	1.384 (4)
F1—C4	1.361 (3)	C20—H20	0.9500
F2—C10	1.369 (3)	C21—C22	1.370 (4)
F3—C16	1.363 (3)	C21—H21	0.9500
F4—C22	1.367 (3)	C22—C23	1.365 (4)
F5—C28	1.362 (3)	C23—C24	1.389 (4)
F6—C34	1.372 (3)	C23—H23	0.9500
O1—N1	1.191 (3)	C24—H24	0.9500
O2—N2	1.197 (3)	C25—C26	1.395 (4)
C1—C2	1.384 (4)	C25—C30	1.402 (4)
C1—C6	1.399 (4)	C26—C27	1.390 (4)
C2—C3	1.396 (4)	C26—H26	0.9500
C2—H2	0.9500	C27—C28	1.367 (4)
C3—C4	1.362 (4)	C27—H27	0.9500
C3—H3	0.9500	C28—C29	1.381 (4)
C4—C5	1.375 (4)	C29—C30	1.378 (4)
C5—C6	1.381 (4)	C29—H29	0.9500
C5—H5	0.9500	C30—H30	0.9500
C6—H6	0.9500	C31—C32	1.390 (4)
C7—C12	1.393 (4)	C31—C36	1.399 (4)
C7—C8	1.394 (4)	C32—C33	1.383 (4)
C8—C9	1.387 (4)	C32—H32	0.9500
C8—H8	0.9500	C33—C34	1.366 (4)
C9—C10	1.369 (4)	C33—H33	0.9500
C9—H9	0.9500	C34—C35	1.374 (4)
C10—C11	1.371 (4)	C35—C36	1.380 (4)
C11—C12	1.391 (4)	C35—H35	0.9500
C11—H11	0.9500	C36—H36	0.9500
C12—H12	0.9500	Cl1S—C1S	1.763 (3)
C13—C14	1.383 (4)	Cl2S—C1S	1.753 (3)
C13—C18	1.392 (4)	Cl3S—C1S	1.759 (3)
C14—C15	1.395 (4)	C1S—H1S	1.0000
			
N2—Fe1—N1	127.02 (11)	C15—C16—C17	123.1 (3)
N2—Fe1—P1	101.53 (9)	C16—C17—C18	117.8 (3)
N1—Fe1—P1	108.49 (8)	C16—C17—H17	121.1
N2—Fe1—P2	105.60 (9)	C18—C17—H17	121.1
N1—Fe1—P2	104.96 (9)	C17—C18—C13	121.1 (3)
P1—Fe1—P2	108.27 (4)	C17—C18—H18	119.5
C13—P1—C1	104.24 (13)	C13—C18—H18	119.5
C13—P1—C7	103.62 (13)	C24—C19—C20	118.5 (3)
C1—P1—C7	101.25 (13)	C24—C19—P2	118.4 (2)
C13—P1—Fe1	119.13 (10)	C20—C19—P2	123.1 (2)
C1—P1—Fe1	117.95 (9)	C21—C20—C19	120.7 (3)
C7—P1—Fe1	108.36 (9)	C21—C20—H20	119.6
C19—P2—C25	103.27 (12)	C19—C20—H20	119.6
C19—P2—C31	103.37 (13)	C22—C21—C20	118.4 (3)
C25—P2—C31	103.19 (12)	C22—C21—H21	120.8
C19—P2—Fe1	117.89 (10)	C20—C21—H21	120.8
C25—P2—Fe1	118.31 (9)	C23—C22—F4	118.2 (3)
C31—P2—Fe1	108.92 (9)	C23—C22—C21	123.4 (3)
O1—N1—Fe1	177.0 (2)	F4—C22—C21	118.4 (3)
O2—N2—Fe1	178.1 (2)	C22—C23—C24	117.8 (3)
C2—C1—C6	118.7 (3)	C22—C23—H23	121.1
C2—C1—P1	121.9 (2)	C24—C23—H23	121.1
C6—C1—P1	119.4 (2)	C23—C24—C19	121.2 (3)
C1—C2—C3	121.1 (3)	C23—C24—H24	119.4
C1—C2—H2	119.5	C19—C24—H24	119.4
C3—C2—H2	119.5	C26—C25—C30	118.5 (3)
C4—C3—C2	118.0 (3)	C26—C25—P2	123.1 (2)
C4—C3—H3	121.0	C30—C25—P2	118.4 (2)
C2—C3—H3	121.0	C27—C26—C25	120.7 (3)
F1—C4—C3	119.0 (3)	C27—C26—H26	119.6
F1—C4—C5	117.9 (3)	C25—C26—H26	119.6
C3—C4—C5	123.1 (3)	C28—C27—C26	118.3 (3)
C4—C5—C6	118.3 (3)	C28—C27—H27	120.8
C4—C5—H5	120.8	C26—C27—H27	120.8
C6—C5—H5	120.8	F5—C28—C27	118.8 (3)
C5—C6—C1	120.8 (3)	F5—C28—C29	118.0 (3)
C5—C6—H6	119.6	C27—C28—C29	123.2 (3)
C1—C6—H6	119.6	C30—C29—C28	117.9 (3)
C12—C7—C8	119.1 (3)	C30—C29—H29	121.0
C12—C7—P1	124.2 (2)	C28—C29—H29	121.0
C8—C7—P1	116.6 (2)	C29—C30—C25	121.3 (3)
C9—C8—C7	120.9 (3)	C29—C30—H30	119.4
C9—C8—H8	119.6	C25—C30—H30	119.4
C7—C8—H8	119.6	C32—C31—C36	118.9 (3)
C10—C9—C8	118.0 (3)	C32—C31—P2	124.2 (2)
C10—C9—H9	121.0	C36—C31—P2	116.9 (2)
C8—C9—H9	121.0	C33—C32—C31	120.6 (3)
F2—C10—C9	118.3 (3)	C33—C32—H32	119.7
F2—C10—C11	118.3 (3)	C31—C32—H32	119.7
C9—C10—C11	123.4 (3)	C34—C33—C32	118.4 (3)
C10—C11—C12	118.2 (3)	C34—C33—H33	120.8
C10—C11—H11	120.9	C32—C33—H33	120.8
C12—C11—H11	120.9	C33—C34—F6	118.7 (3)
C11—C12—C7	120.4 (3)	C33—C34—C35	123.3 (3)
C11—C12—H12	119.8	F6—C34—C35	117.9 (3)
C7—C12—H12	119.8	C34—C35—C36	117.9 (3)
C14—C13—C18	119.2 (3)	C34—C35—H35	121.0
C14—C13—P1	123.7 (2)	C36—C35—H35	121.0
C18—C13—P1	117.0 (2)	C35—C36—C31	120.8 (3)
C13—C14—C15	120.6 (3)	C35—C36—H36	119.6
C13—C14—H14	119.7	C31—C36—H36	119.6
C15—C14—H14	119.7	Cl2S—C1S—Cl3S	110.41 (17)
C16—C15—C14	118.1 (3)	Cl2S—C1S—Cl1S	110.44 (17)
C16—C15—H15	121.0	Cl3S—C1S—Cl1S	111.10 (17)
C14—C15—H15	121.0	Cl2S—C1S—H1S	108.3
F3—C16—C15	118.5 (3)	Cl3S—C1S—H1S	108.3
F3—C16—C17	118.3 (3)	Cl1S—C1S—H1S	108.3
			
N2—Fe1—P1—C13	82.58 (13)	P1—C13—C14—C15	−178.0 (3)
N1—Fe1—P1—C13	−141.68 (13)	C13—C14—C15—C16	0.1 (5)
P2—Fe1—P1—C13	−28.29 (11)	C14—C15—C16—F3	−179.9 (3)
N2—Fe1—P1—C1	−149.51 (13)	C14—C15—C16—C17	0.2 (5)
N1—Fe1—P1—C1	−13.77 (13)	F3—C16—C17—C18	−179.3 (3)
P2—Fe1—P1—C1	99.61 (10)	C15—C16—C17—C18	0.7 (5)
N2—Fe1—P1—C7	−35.38 (12)	C16—C17—C18—C13	−1.7 (4)
N1—Fe1—P1—C7	100.36 (13)	C14—C13—C18—C17	2.0 (4)
P2—Fe1—P1—C7	−146.25 (10)	P1—C13—C18—C17	179.1 (2)
N2—Fe1—P2—C19	−175.56 (13)	C25—P2—C19—C24	−88.2 (2)
N1—Fe1—P2—C19	48.24 (13)	C31—P2—C19—C24	164.5 (2)
P1—Fe1—P2—C19	−67.47 (11)	Fe1—P2—C19—C24	44.3 (2)
N2—Fe1—P2—C25	−50.12 (13)	C25—P2—C19—C20	89.7 (2)
N1—Fe1—P2—C25	173.69 (13)	C31—P2—C19—C20	−17.6 (3)
P1—Fe1—P2—C25	57.98 (11)	Fe1—P2—C19—C20	−137.8 (2)
N2—Fe1—P2—C31	67.18 (13)	C24—C19—C20—C21	−0.9 (4)
N1—Fe1—P2—C31	−69.01 (12)	P2—C19—C20—C21	−178.8 (2)
P1—Fe1—P2—C31	175.28 (9)	C19—C20—C21—C22	0.4 (4)
C13—P1—C1—C2	112.3 (2)	C20—C21—C22—C23	0.0 (5)
C7—P1—C1—C2	−140.4 (2)	C20—C21—C22—F4	179.6 (3)
Fe1—P1—C1—C2	−22.4 (3)	F4—C22—C23—C24	−179.5 (2)
C13—P1—C1—C6	−67.8 (2)	C21—C22—C23—C24	0.1 (5)
C7—P1—C1—C6	39.5 (2)	C22—C23—C24—C19	−0.5 (4)
Fe1—P1—C1—C6	157.51 (19)	C20—C19—C24—C23	0.9 (4)
C6—C1—C2—C3	0.3 (4)	P2—C19—C24—C23	179.0 (2)
P1—C1—C2—C3	−179.7 (2)	C19—P2—C25—C26	7.5 (3)
C1—C2—C3—C4	−0.7 (4)	C31—P2—C25—C26	114.9 (2)
C2—C3—C4—F1	−178.7 (2)	Fe1—P2—C25—C26	−124.8 (2)
C2—C3—C4—C5	0.5 (4)	C19—P2—C25—C30	−173.6 (2)
F1—C4—C5—C6	179.2 (2)	C31—P2—C25—C30	−66.2 (2)
C3—C4—C5—C6	0.1 (4)	Fe1—P2—C25—C30	54.1 (2)
C4—C5—C6—C1	−0.4 (4)	C30—C25—C26—C27	0.2 (4)
C2—C1—C6—C5	0.2 (4)	P2—C25—C26—C27	179.1 (2)
P1—C1—C6—C5	−179.7 (2)	C25—C26—C27—C28	1.2 (4)
C13—P1—C7—C12	−0.2 (3)	C26—C27—C28—F5	179.2 (3)
C1—P1—C7—C12	−108.0 (3)	C26—C27—C28—C29	−1.2 (5)
Fe1—P1—C7—C12	127.2 (2)	F5—C28—C29—C30	179.3 (3)
C13—P1—C7—C8	−178.8 (2)	C27—C28—C29—C30	−0.3 (5)
C1—P1—C7—C8	73.3 (2)	C28—C29—C30—C25	1.8 (4)
Fe1—P1—C7—C8	−51.4 (2)	C26—C25—C30—C29	−1.8 (4)
C12—C7—C8—C9	1.3 (4)	P2—C25—C30—C29	179.3 (2)
P1—C7—C8—C9	−180.0 (2)	C19—P2—C31—C32	103.9 (2)
C7—C8—C9—C10	−2.5 (4)	C25—P2—C31—C32	−3.4 (3)
C8—C9—C10—F2	−178.0 (3)	Fe1—P2—C31—C32	−130.0 (2)
C8—C9—C10—C11	2.1 (5)	C19—P2—C31—C36	−76.6 (2)
F2—C10—C11—C12	179.7 (2)	C25—P2—C31—C36	176.1 (2)
C9—C10—C11—C12	−0.4 (5)	Fe1—P2—C31—C36	49.6 (2)
C10—C11—C12—C7	−0.9 (4)	C36—C31—C32—C33	0.0 (4)
C8—C7—C12—C11	0.4 (4)	P2—C31—C32—C33	179.5 (2)
P1—C7—C12—C11	−178.2 (2)	C31—C32—C33—C34	0.7 (4)
C1—P1—C13—C14	−8.4 (3)	C32—C33—C34—F6	178.3 (2)
C7—P1—C13—C14	−114.0 (3)	C32—C33—C34—C35	−0.4 (4)
Fe1—P1—C13—C14	125.6 (2)	C33—C34—C35—C36	−0.5 (4)
C1—P1—C13—C18	174.7 (2)	F6—C34—C35—C36	−179.3 (2)
C7—P1—C13—C18	69.1 (2)	C34—C35—C36—C31	1.2 (4)
Fe1—P1—C13—C18	−51.3 (2)	C32—C31—C36—C35	−0.9 (4)
C18—C13—C14—C15	−1.2 (5)	P2—C31—C36—C35	179.5 (2)
